# Associating Gait Phase and Physical Fitness with Global Cognitive Function in the Aged

**DOI:** 10.3390/ijerph17134786

**Published:** 2020-07-03

**Authors:** Byungjoo Noh, Changhong Youm, Myeounggon Lee, Hwayoung Park

**Affiliations:** 1 Department of Health Care and Science, College of Health Sciences, Dong-A University, Saha-gu, Busan 49315, Korea; bnoh@dau.ac.kr; 2 Biomechanics Laboratory, College of Health Sciences, Dong-A University, Saha-gu, Busan 49315, Korea; freestyle710@naver.com (M.L.); app00113@dau.ac.kr (H.P.)

**Keywords:** aging, dementia, cognitive decline, physical activity, inertial measurement unit

## Abstract

This study aimed to identify classifier variables by considering both gait and physical fitness for identifying adults aged over 75 years and global cognitive function declines in older adults. The participants included 735 adults aged 65–89 years who were asked to walk at three different speeds (slower, preferred, and faster) while wearing inertial measurement units embedded in shoe-type data loggers and to perform nine physical fitness tests. The variability in the stance phase as well as the strength, balance, and functional endurance showed a strong dependence on the age being over 75 years. The cognitive function was evaluated by the Mini-Mental State Examination; a longer stance phase at a slower walking speed and decreased grip strength and five times sit-to-stand were associated with cognitive function. These findings may be useful for determining the decline in physical performance of older adults. A longer stance phase and decreased grip strength and five times sit-to-stand may be factors that help distinguish declines in cognitive function from normal age-related declines.

## 1. Introduction

Dementia is a serious health problem that can impact the physical health and quality of life of older adults [1]. The decline in cognitive function with age, which can lead to mild cognitive impairment and dementia, has been associated with functional limitations and disability [2]. This may be because motor performance is longitudinally associated with cognitive impairment [3] and dementia [4,5]. Furthermore, impairment of the motor system such as gait abnormality and a low level of physical fitness precede the onset of cognitive decline with age [3,4,5] or during the early stages of dementia [6]. Gait performance and cognitive function have shown distinct patterns of association, such as executive functions with gait variability [7,8] and memory with the phase domain [8]. Moreover, slow walking is a significant predictor of cognitive decline or the risk of dementia [9,10]. Our previous study showed that decreased global cognitive function in older women can be associated with gait abnormalities such as increased gait variability and the phase domain at different gait speeds [11]. Gait abnormality may not only increase the risk of dementia in older adults but also indicate a low level of physical fitness in domains [5] such as mobility [12], balance [13], strength [14], and aerobic fitness [15]. It is not difficult to find a relationship between physical fitness, including mobility, and cognitive function in older adults [16].

A limited study investigated the phase domain for assessing cognitive function declines; lower global cognitive function showed the strongest relationship with a longer stance phase in older adults aged over 75 years [11,17], which could lead to falling [18]. This effect may be aggravated in older adults aged over 75 years because physiological changes may occur above this age threshold [19]. In addition, some studies performed multiple physical performance tests for identifying physical functions associated with cognitive functions [20,21,22]. Studies of physical performance characteristics for assessing declines in cognitive function remain necessary for identifying at different approaches with several gait variables (not only gait speed) with numerous consecutive steps and physical fitness variables. Further, the relations of both gait and physical fitness domains with declines in cognitive function should be investigated. In this light, the present study aims to identify the following:

- Classifier variables for identifying declines in physical function in adults aged over 75 years by assessing gait and physical fitness variables;
- Domain parameters of the gait at three different walking speeds and physical fitness that are strongly associated with lower global cognitive function in older adults.

## 2. Materials and Methods

### 2.1. Participants

**Figure 1:**
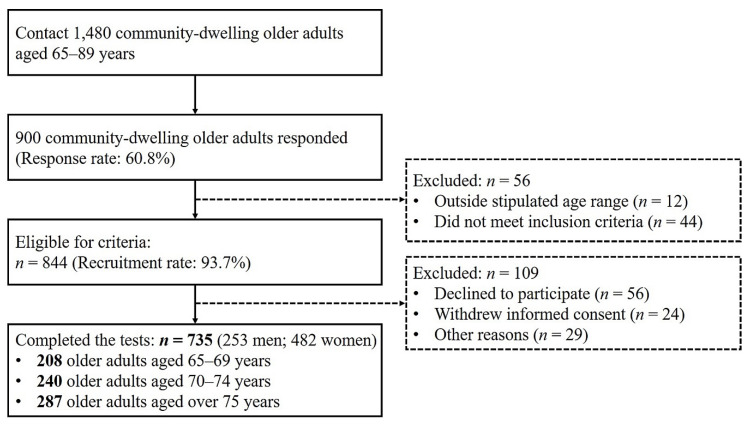
Flow diagram for participant recruitment.

This study was conducted as part of a community-wide survey in Busan Metropolitan City in 2018–2019. We recruited 844 participants aged 65–89 years through advertisements placed in the local community (a power > 80%). The inclusion criteria for participants were as follows: (a) age of 65–89 years, (b) no musculoskeletal injuries, and (c) no history of neurological problems within the last 6 months. In total, 735 participants took part in the over-ground walking and physical fitness tests (Figure 1). All participants provided written informed consent. This study was approved by the Institutional Review Board of Dong-A University (IRB number: 2–1040709–AB–N–01–201808–HR–023–02).

### 2.2. Instrumentation

Data were collected with a gait analysis system (DynaStab^TM^, JEIOS, Busan, South Korea) comprising shoe-type data loggers (Smart Balance SB-1^®^, JEIOS, Busan, South Korea). The data logger was embedded with Bluetooth inertial measurement unit (IMU) sensors (IMU-3000^TM^, InvenSense, San Jose, CA, USA) on both side outsoles. Tri-axial acceleration (up to ± 6 *g*) and tri-axial angular velocities (up to ±500° s^−1^) along three orthogonal axes were sampled at a sampling frequency of 100 Hz using a data acquisition system (Smart Balance version 1.5, JEIOS, Busan, South Korea) [23,24]. The IMU sensors were constructed using a local coordinate system with anterior/posterior, medial/lateral, and vertical coordinates [23].

### 2.3. Test Procedures

Prior to the over-ground walking test and physical fitness test, biometric data (InBody 270, Biospace, Seoul, South Korea) of all participants were recorded. Participants were asked to answer the habitual physical activity (PA) questionnaire. Habitual physical activity levels were evaluated with the use of the international physical activity questionnaire–short form, which pertained to the self-reported physical activity of the participants. The total metabolic equivalents (MET-min/week) were calculated based on this questionnaire [25]. Participants were verbally instructed with a visual demonstration to perform over-ground walking for 10 min as a familiarization.

#### 2.3.1. Global Cognitive Function

The Mini-Mental State Examination (MMSE) was used to assess global cognitive function; this is a widely used test of global cognitive function and screening tool for dementia [26]. Participants were answered a 30-point questionnaire that includes tests pertaining to orientation, attention, memory, language, and visual-spatial skills. Participants were categorized as having normal cognitive function if they had an MMSE score of 24 or greater and having abnormal cognitive function if they scored below 24. For evaluations, the MMSE score was normalized by the Z-score ((value–mean)/standard deviation) because it is not supposed to be used as a continuous variable.

#### 2.3.2. Physical Fitness Test

We assessed four domains of physical fitness: strength (upper/lower body), flexibility (upper/lower body), balance (static/dynamic), and functional (or cardiorespiratory) endurance. All participants completed nine physical fitness tests in the following order (see Table S1):

1. Grip strength (dominant hand) was measured with an isometric digital handgrip dynamometer (TKK 5401 Grip-D, Takei Scientific Instruments, Tokyo, Japan) to assess the upper body strength;
2. Bicep curls were performed with a dumbbell (3 kg for men, 2 kg for women) to assess the upper body strength;
3. Sit-to-stand was performed five times to assess the lower body strength;
4. Standing time (ST) from a long sitting position (LSP) was measured to assess the lower body strength;
5. Back scratching to assess the upper body flexibility;
6. Chair sit and reach to assess the lower body flexibility;
7. Single-leg balance (dominant leg) to assess the static balance;
8. A 3 m timed-up-and-go test to assess the dynamic balance; and
9. A 6 min walk test to assess the functional (or cardiorespiratory) endurance.

The mean scores were calculated for two attempts of each physical fitness test. For further analysis, the scores of each physical fitness test were normalized into Z-scores of all variables.

#### 2.3.3. Over-Ground Walking Test at Different Speeds

The over-ground walking test along a straight 20 m walkway at 80% of usual (slower), usual walking (preferred), and 120% of usual (faster) speeds were performed three times. The preferred speed is normally used in daily activities without any support during over-ground walking. The 20% slower and faster walking speed was calculated relative to the preferred speed [27], which were quantified using a metronome (beats/min). Participants were asked to perform the over-ground walking test as close as possible to the target slower and faster walking speed paced by a metronome [11]. The participants completed the over-ground walking test wearing the shoe-type data loggers with multiple shoe sizes to fit the tested individuals.

### 2.4. Data Analysis

The over-ground walking data (see Supplementary Materials Table S2: Raw data) from a gait analysis system were passed through a second-order Butterworth low-pass filter with a cutoff frequency of 10 Hz [23,24]. We excluded the two initial steps (acceleration) and two final steps at the end of the test (deceleration) for analyzing the steady-state condition. Heel strikes and toe-offs were identified that occurred when the linear acceleration along the antero-posterior axis and the vertical axis reached its maximum value during a gait cycle, respectively [23,24]. The stance phase as a phase domain and the coefficient of variance (CV, (standard deviation/mean) × 100) values for the stance phase as a gait variability were calculated. The gait variable was normalized to Z-scores for all variables.

#### Covariates

The covariates included in the analysis were age, sex, body mass index, level of education, and PA level. The level of education was defined as a categorical variable (elementary school education or less, middle school education, high school education, college degree, or higher).

### 2.5. Statistical Analyses

The Shapiro–Wilk test was used to examine whether a variable is normally distributed. An independent sample t-test was performed to compare differences in demographics, MMSE score, gait parameters at the three different walking speeds, and physical fitness parameters. Binary logistic regression was conducted to determine classifiers for identifying the declined gait abilities and physical fitness of participants aged over 75 years at each walking speed and the physical fitness domain, which contained all confounders. The logistic regression model results were reported in terms of the odds ratio (OR) and 95% confidence interval (CI). Subsequently, stepwise multivariable linear regression analysis was performed to identify the independent variables (each gait speed and physical fitness domains) and explain the significance of the dependent variables (MMSE scores). All models used the MMSE scores, gait variables (stance phase and CV of stance phase at three different speeds), and physical fitness variables (grip strength, biceps curls, five times sit-to-stand, ST from an LSP, back scratching, chair sit and reach, single-leg balance, 3 m timed up-and-go, and 6 min walk). The analyses were conducted by using SPSS for Windows (version 25.0, IBM Corp., Armonk, NY, USA), and the level of significance was *p* < 0.05.

## 3. Results

### 3.1. Demographics and Characteristics of Physical Performance

Table 1 presents the demographic, gait, and physical fitness characteristics of the 735 participants included in the study. The participants had an average age of 73.1 ± 5.1 years (range: 65–89 years). The men had greater physical activity (*p* < 0.001), more years of education (*p* < 0.001), and higher MMSE scores (*p* = 0.023) than the women. With respect to the gait variables, the men showed a lower stance phase than the women at all three walking speeds (*p* < 0.001). With respect to the physical fitness variables, the men performed better than the women at the grip strength, bicep curls, five-time sit-to-stand, ST from an LSP, 3 m timed-up-and-go, 6-min walk, lower back scratching, and chair sit and reach (all *p* < 0.001).

### 3.2. Gait Classifiers with Three Different Speed and Physical Fitness Variables to Identify Participants Aged Above 75 Years

Table 2 summarizes only the statistically significant results from the binary logistic regression for all participants. The binary logistic regression models for identifying participants over 75 years by gait and physical fitness variables showed that the ORs were significantly different for the MMSE score (OR: 0.605; *p* < 0.001) and CV of the stance phase at the preferred speed (OR: 1.626; *p* = 0.024) in the over-ground walking test. In addition, the ORs were significantly different for the grip strength (OR: 0.541; *p* < 0.001), single-leg balance (OR: 0.491; *p* < 0.001), and 6-min walk (OR: 0.451; *p* < 0.001) in the physical fitness test.

### 3.3. Association of Gait with the MMSE Score

Table 3 lists only the statistically significant results for the association of gait at three different speeds and physical fitness variables with the MMSE score of older adults. After adjustments to the confounders, the stance phase at the slower speed (β = 0.088, *p* = 0.023), grip strength (β = 0.148, *p* < 0.001), and five times sit-to-stand (β = −0.111, *p* = 0.003) showed a significant association with the MMSE score.

## 4. Discussion

This study demonstrated classifier variables for participants over 75 years and a sensitive assessment for identifying declines in global cognitive function in older adults. The key findings are as follows:

1. The gait and nine physical fitness variables indicated that the variability in the stance phase and the strength, balance, and functional endurance showed a strong dependence on the age being over 75 years.
2. The stance phase at the slower walking speed and grip strength and five times sit-to-stand were associated with the global cognitive function in older adults.

### 4.1. Gait and Physical Fitness Domain Parameters Reflecting Age over 75 Years

With respect to gait variables, higher gait variability; lower strength, static balance, and functional endurance; and lower cognitive function showed a strong dependence on the age being over 75 years. Our findings are consistent with those of previous studies reporting that the variability domain for women aged over 75 years [11] and physical fitness [28] are highly dependent on aging. This relationship may be related to increased stride-to-stride fluctuations during walking because of greater foot contact time on the ground from decreased cognitive function. This may lead to dynamic gait instability in individuals aged over 75 years, who have a lower static balance than individuals aged below 75 years. Furthermore, lower muscular strength may increase gait variability responses [29]. This is supported by the finding that decreased dynamic gait stability is associated with less steady force output during walking, which depends on a decline in motor control and the automatic stepping mechanism [30,31]. In addition, 78 years may be the threshold age for when physiological changes occur in the human plasma proteome [19]. Therefore, our results may identify parameters for motor changes at the threshold age of 75 years. Gait variability, muscular strength, balance, and functional endurance should be useful parameters for identifying changes in motor patterns of the aged. Physical performances are more vulnerable in women. For example, the 3 m timed-up-and-go test showed that the women had a longer stance phase and worse dynamic balance than the men. The female disadvantage has been well-established among older adults: women have a higher life expectancy but, ironically, perform worse [32,33] because of less physical activity and walking [34]. Further, morphological differences in sex, such as anatomy and physiology, affect the neuromuscular performance [29,35,36]. Women have smaller skeletal muscles and a lower percentage of type II muscle fibers [36,37] because of sex-related differences in human skeletal muscle gene expression and interactions with sex-specific hormones [38,39].

### 4.2. Identifying Cognitive Function Decline According to Gait and Physical Fitness Variables

Previous studies have presented similar findings: an association among a longer stance phase, lower strength (grip strength and five times sit-to-stand), and cognitive function decline in older adults [8,11,14,17]. In contrast, biceps curl and ST from an LSP did not show any significant association with cognitive function. Biceps curls involve repetitive arm movements with light loading (men: 3 kg; women 2 kg) that is close to the muscle endurance. A longer stance phase in aged individuals with cognitive function decline may contribute to a decreasing walking speed and manifest owing to inadequate propulsive force. The optimal propulsive force generation should be achieved during the stance phase [11]. This inadequate propulsive force generation may also be due to reduced muscular strength based on the results of the physical fitness test. Previous studies’ results supported an association between lower upper- and lower-body muscular strength and lower cognitive function in older adults [40,41]. This may be linked to the decreased volumes of gray and white matter in multiple brain regions and white matter hyperintensities [42]. Furthermore, dynamic stability is affected by a longer stance phase during walking. This factor would also increase the risk of falling in aged individuals with cognitive function decline. Indeed, a longer stance phase during walking could be used to distinguish cognitive function decline from normal age-related decline.

Our over-ground walking test with modified gait speeds, especially the slower speed, may have served as a dual-task. The stance phase at the slower walking speed showed an association with the global cognitive function. Gait speed modification, which is the volitional control of movement, may be generated in response to supraspinal inputs but not owing to underlying locomotor patterns by a network of spinal interneurons (commonly referred to as the central pattern generator (CPG)) at the spinal cord level [43,44]. Furthermore, as they age, older adults become less peripherally aware during walking and less sensitive to peripheral reflexes with more reliance on central regulation [45]. Song and Geyer suggested that feedback integration may be functionally more important than the CPG to human locomotion [46]. Therefore, a walking test with speed modification could be a suitable method for assessing cognitive function decline in older adults. We suggest that cognitive function may be improved in older adults through approaches such as physical activities or modifying the walking speed based on auditory feedback, which can help evoke supraspinal inputs.

Previous studies have reported that slow walking speed is associated with cognitive function decline [47]. However, walking speed was not considered as a gait variable in the study, even though a reduced walking speed may be effective variable for predicting a decline in cognitive functions. Nevertheless, in the past decades, walking speed has been useful to assess the health status (e.g., frailty and dementia) in clinical utilities. However, in recent year, there has been a high demand for new approaches that use low-cost-IMU-sensors-embedded gait assessment system to assess health status [48]. Specifically, clinical and research settings can use this system to precisely assess the health status with several gait variables (e.g., phases, variability, and gait asymmetry). Therefore, we considered the over-ground walking test with consecutive steps to strengthen the reliability of the gait variables.

Our findings must be considered within the limitations of the study. First, the over-ground walking test was not directly conducted as a dual-task (i.e., combined motor and cognitive tasks) to measure the associations between motor and cognitive functions. Therefore, the stance phase at faster walking speed may not show a significant association with cognitive function. However, gait speed modification may obtain the effects of a dual-task because gait analysis under various walking speed conditions may be challenging for individuals with cognitive function decline [31]. Therefore, gait speed modification may help with determining cognitive function decline through motor functions. Second, our findings cannot exclude the possibility of sampling bias for sex (482 older women vs. 253 older men). Women yielded slower walking speeds with shorter step lengths and worse muscle strength than men; this pattern may affect the results observed in the physical performance, even though the models were adjusted for sex. Third, the over-ground walking test was conducted with an IMU-based gait analysis system. Even though this system has been validated for the motion capture of patients with Parkinson’s disease, further validation should be performed in various environments and with subjects having various variables. Fourth, this study was based on cross-sectional rather than longitudinal data. Further studies should follow the same sample of older adults over time. Fifth, the ST from an LSP, which was included to reflect older adults’ tendency to sit on the floor in Asian cultures, was not associated with cognitive function despite five times sit-to-stand was associated due to the violation of the multicollinearity. Thus, this issue should be considered when interpreting the results. Lastly, the MMSE was only used to assess the global cognitive function. Fundamentally, the MMSE was used as a screening test of global cognitive function or dementia; it could not measure certain cognitive functions although several studies have used it to assess global cognitive function [11,17,49].

## 5. Conclusions

In the study, we found that the stance phase at three different walking speeds and physical fitness levels should be considered as classifier variables to identify gait and physical fitness declines in individuals aged 75 years or older. In addition, declined cognitive function was associated with a longer stance phase at a slower walking speed and decreased grip strength and five times sit-to-stand in older adults. Our results are useful for determining the decline in physical performance of older adults and may potentially be used in clinical environments to measure the effectiveness of interventions meant to prevent or delay cognitive impairment.

## Figures and Tables

**Table 1:** Demographic, gait, and physical fitness characteristics of participants.

Variables	All Participants (*n* = 735)	Older Men (*n* = 253)	Older Women (*n* = 482)	*p*-Value
Age (years)	73.1 ± 5.1	74.2 ± 5.2	72.6 ± 5.0	**<0.001**
Height (cm)	157.3 ± 8.1	165.6 ± 5.9	152.9 ± 5.2	**<0.001**
Body weight (kg)	61.1 ± 8.7	66.7 ± 7.6	58.3 ± 7.9	**<0.001**
Body mass index (kg/m^2^)	24.7 ± 2.9	24.3 ± 2.4	24.9 ± 3.1	**0.003**
Total PA (MET-min/week)	1873.7 ± 1792.7	2225.2 ± 1771.7	1689.2 ± 1777.8	**<0.001**
**Education (years)**	9.1 ± 3.9	10.5 ± 4.0	8.4 ± 3.7	**<0.001**
0 year	36 (4.9%)	7 (2.8%)	29 (6.0%)	
1–6 years	249 (33.9%)	57 (22.5%)	192 (39.8%)	
7–12 years	373 (50.7%)	142 (56.1%)	231 (47.9%)	
>13 years	77 (10.5%)	47 (18.5%)	30 (6.2%)	
**MMSE score**	26.4 ± 2.9	26.8 ± 2.7	26.3 ± 3.0	**0.023**
<24	100 (13.6%)	27 (10.7%)	73 (15.1%)	
**Slower speed**				
Stance phase (%)	59.2 ± 1.6	58.9 ± 1.6	59.4 ± 1.6	**<0.001**
CV of stance phase (%)	4.5 ± 2.2	4.4 ± 2.2	4.6 ±2.2	0.279
**Preferred speed**				
Stance phase (%)	57.5 ± 1.7	57.1 ± 1.7	57.7 ± 1.7	**<0.001**
CV of stance phase (%)	3.0 ± 1.7	3.1 ± 1.7	2.9 ± 1.7	0.379
**Faster speed**				
Stance phase (%)	55.7 ± 1.8	55.2 ± 1.9	55.9 ± 1.8	**<0.001**
CV of stance phase (%)	2.4 ± 1.3	2.3 ± 1.3	2.4 ± 1.3	0.691
**Physical fitness**				
Grip strength (kg)	25.9 ± 7.4	33.4 ± 6.1	22.0 ± 4.3	**<0.001**
Biceps curl (no. of reps.)	25.9 ± 7.7	28.5 ± 8.2	24.6 ± 7.1	**<0.001**
Five times sit-to-stand (s)	9.7 ± 3.7	9.1 ± 3.2	10.0 ± 3.9	**<0.001**
ST from an LSP (s)	3.6 ± 2.1	3.2 ± 1.8	3.8 ± 2.2	**<0.001**
Back scratch (cm)	−12.7 ± 13.5	−20.1 ± 14.0	−8.8 ± 11.5	**<0.001**
Chair sit and reach (cm)	18.4 ± 10.5	11.3 ± 10.8	22.1 ± 8.2	**<0.001**
Single-leg balance (s)	18.2 ± 20.3	17.9 ± 19.1	18.4 ± 20.9	0.735
3 m timed-up-and-go (s)	7.8 ± 2.3	7.2 ± 2.3	8.1 ± 2.2	**<0.001**
6-min walk (m)	463.6 ± 105.6	495.8 ± 110.4	446.7 ± 99.0	**<0.001**

Mean ± SD: mean and standard deviation; PA: physical activity; METs: metabolic equivalents; MMSE: mini-mental state examination; CV: coefficient of variance; ST: standing time; LSP: long sitting position; boldface denotes a significant difference between older men and women; significant difference: *p* < 0.05.

**Table 2:** Summary of binary logistic regression model for identifying participants aged over 75 years.

Predictors	β (SE)	Odds Ratio	95% CI	*p*-Value
**MMSE score**	−0.503 (0.092)	0.605	0.505–0.724	<0.001
**Over-ground walking**				
CV of stance phase (preferred)	0.486 (0.216)	1.626	1.065–2.483	0.024
**Physical fitness**				
Grip strength	−0.614 (0.162)	0.541	0.394–0.744	<0.001
Single-leg balance	−0.712 (0.138)	0.491	0.375–0.642	<0.001
6-min walk	−0.797 (0.173)	0.451	0.321–0.633	<0.001

Model adjusted for sex, body mass index, education level, and physical activity level. SE: standard error; CI: confidence interval; MMSE: mini-mental state examination; CV: coefficient of variance; significant difference: *p* < 0.05.

**Table 3:** Association of gait at three different speeds and physical fitness variables with global cognitive function in older adults.

Variables	MMSE Score		
	β (SE)	*t*	*p*-Value
**Overground walking**	***R*^2^ = 0.191**		
Stance phase (slow)	0.088 (0.039)	2.276	0.023
**Physical fitness**	***R*^2^ = 0.236**		
Grip strength	0.148 (0.036)	4.076	<0.001
Five times sit-to-stand	−0.111 (0.037)	−3.025	0.003

Model adjusted for age, sex, body mass index, education level, and physical activity level. MMSE: mini-mental state examination; SE: standard error; significant difference, *p* < 0.05.

## Supplementary Materials

The following are available online at https://www.mdpi.com/1660-4601/17/13/4786/s1, Table S1: Physical fitness test protocols, Table S2: Raw data.
