# Supplementary material for: Associating Gait Phase and Physical Fitness with Global Cognitive Function in the Aged
Source: Int J Environ Res Public Health. 2020 Jul 3;17(13):4786. doi: 10.3390/ijerph17134786 (PMC7369886; doi:10.3390/ijerph17134786)
Supplement: Supplementary file 1 [file ijerph-17-04786-s001.zip › ijerph-829279 Table S1 Physical fitness test protocols.docx]

**Table S1. Physical fitness test protocols**

| **Upper body strength** | | | |
| --- | --- | --- | --- |
|  | Purpose | Equipment | Protocol |
| Grip strength | To assess upper body strength | Isometric digital handgrip dynamometer | 1. Subjects stand with their shoulder adducted and neutrally rotated. Elbow is toward the body and flexed at 90 degrees and the forearm and wrist in a neutral position. |
|  |  |  | 2. The handle of the dynamometer is adjusted if required - the base should rest on the first metacarpal (heel of palm), while the handle should rest on the middle of the four fingers. |
|  |  |  | 3. When ready the subjects squeeze the dynamometer with maximum isometric effort, which is maintained for about 5 seconds. No other body movement is allowed. |
|  |  |  | 4. The subjects should be strongly encouraged to give maximum effort. |
| Biceps curls | To assess upper body strength | Dumbbell  (3 kg for men;  2 kg for women) | 1. Investigator asks subjects to perform for 30 seconds, using the stopwatch. |
|  |  |  | 2. Do as many curls as subjects can in the allotted 30‐second time period, moving in a controlled manner. |
|  |  |  | 3. Do a full curl, squeezing lower arm against upper arm at the top of each curl, and returning to a straight arm each time. Keep upper arm still, and do not swing the weight. |
|  |  |  | 4. If subjects have started raising the weight again and are over halfway up when time is called, it may count. |
|  |  |  | 5. Record the score on the scorecard. |

| **Lower body strength** | | | |
| --- | --- | --- | --- |
|  | Purpose | Equipment | Protocol |
| Five times  sit-to-stand | To assess lower body strength | Chair without arms, Stopwatch | 1. Place the chair against a wall where it should be stable. |
|  |  |  | 2. Sit in the middle of the chair with feet flat on the floor, shoulder-width apart, back straight. |
|  |  |  | 3. Cross arms at the wrist and place them against the chest. |
|  |  |  | 4. Do rise to a full stand and sit again as many times as subjects can during the 30‐second interval. |
| Standing time from a long sitting position | To assess lower body strength | Stopwatch | 1. Subjects sit on the floor with knees extended. |
|  |  |  | 2. Subjects stand up as quickly as possible to a stable erect position from a long sitting position on the floor. |
|  |  |  | 3. Record the time on the scorecard. |

| **Upper body flexibility** | | | |
| --- | --- | --- | --- |
|  | Purpose | Equipment | Protocol |
| Back scratching | To assess upper body flexibility | Ruler | 1. Place left arm straight up in the air left shoulder. |
|  |  |  | 2. Bend left arm at the elbow to reach toward the back, with fingers extended. Elbow pointed toward the ceiling. |
|  |  |  | 3. Place right hand behind back with palm out and fingers extended up. |
|  |  |  | 4. Reach up as far as possible and attempt to touch the fingers of two hands together. Some people are not able to touch at all, while others’ fingers may overlap. |
|  |  |  | 5. The distance between the fingertips of one hand and the other is measured to the nearest half‐inch. If the subject’s fingers overlap, the amount of the overlap is measured. |
|  |  |  | 6. Take stretches with each arm, determining which side is more ­flexible. Recording only the most ­flexible side. Fingertips just touching receive a score of “0”. |

| **Lower body flexibility** | | | |
| --- | --- | --- | --- |
|  | Purpose | Equipment | Protocol |
| Chair  sit and reach | To assess lower body flexibility | Chair, Ruler | 1. Place the chair against a wall where it should be stable. |
|  |  |  | 2. Slide forward in the chair until subjects are able to straighten one of their legs. The ankle of the straight leg should be flexed at about a 90‐degree angle. The other foot should be flat on the floor. |
|  |  |  | 3. Place one of the hands directly on top of the other so that subjects are stacked with their fingers extended. |
|  |  |  | 4. Exhale as subject bend forward at the hip and try to reach their toes. If the extended leg begins to bend, move back in the chair until the leg is straight. |
|  |  |  | 5. Hold the stretch for at least 2 seconds and do not bounce or jerk as reach. |
|  |  |  | 6. Take reaches on each leg, determining which side is more -flexible. Recording only the most flexible side. The center of the toe of the shoe is considered to be a measurement of “0”. |

| **Static balance** | | | |
| --- | --- | --- | --- |
|  | Purpose | Equipment | Protocol |
| Single-leg balance | To assess static balance | Stopwatch | 1. Performed with eyes open and arms on the hips. |
|  |  |  | 2. Subjects must stand unassisted on one leg and is timed in seconds from the time one foot is flexed off the floor to the time when it touches the ground or the standing leg, or an arm leaves the hips. |
|  |  |  | 3. Record the time on the scorecard. |

| **Dynamic balance** | | | |
| --- | --- | --- | --- |
|  | Purpose | Equipment | Protocol |
| 3 m timed up and go | To assess dynamic balance | Chair, Cone, Stopwatch | 1. Sit in the chair with hands-on thighs, feet flat on the floor with one foot slightly ahead of the other. |
|  |  |  | 2. Investigator signals, “go” and start the watch. For test accuracy, the investigator must start the watch on the signal, “go.” Do not wait to start the watch after subjects have started to move. |
|  |  |  | 3. The test is timed to the nearest tenth (0.1) of a second, so it is important to be as accurate as possible when starting and stopping the watch. |
|  |  |  | 4. Upon the signal “go” rise from the chair and walk as quickly as possible out to the marker. Subjects may press off their thighs of the chair when they rise. Do not run. Walk around the outside of the marker and return to their seat as quickly as possible, being sure to be safe in their movements. |
|  |  |  | 5. As soon as they are fully seated again, the investigator should stop the watch and record their time to the nearest tenth of a second. |

| **Functional (or cardiorespiratory) endurance** | | | |
| --- | --- | --- | --- |
|  | Purpose | Equipment | Protocol |
| 6 min walk | To assess functional (or cardiorespiratory) endurance | Chair, Cone, Stopwatch, Measurement scale | 1. Place cones at either end of the 30 m stretches as turning points. |
|  |  |  | 2. The subjects are to walk as far as possible for 6 minutes. They walk back in the hallway. |
|  |  |  | 3. Six minutes is a long time to walk, so they are exerting themselves. Subjects are permitted to slow down, to stop, and to rest as necessary. They may lean against the wall while resting, but resume walking as soon as they are able. |
|  |  |  | 4. Subjects should pivot briskly around the cones and continue back the other way without hesitation. |
|  |  |  | 5. Measure the best distance walked in the meter when timed out. |
